# Krüppel-Like Factor KLF8 Plays a Critical Role in Adipocyte Differentiation

**DOI:** 10.1371/journal.pone.0052474

**Published:** 2012-12-21

**Authors:** Haemi Lee, Hyo Jung Kim, Yoo Jeong Lee, Min-Young Lee, Hyeonjin Choi, Hyemin Lee, Jae-woo Kim

**Affiliations:** 1 Department of Biochemistry and Molecular Biology, Integrated Genomic Research Center for Metabolic Regulation, Institute of Genetic Science, Yonsei University College of Medicine, Seoul, Korea; 2 Brain Korea 21 Project for Medical Science, Yonsei University, Seoul, Korea; 3 Department of Integrated OMICS for Biomedical Sciences, WCU Program of Graduate School, Yonsei University, Seoul, Korea; Wageningen University, The Netherlands

## Abstract

KLF8 (Krüppel-like factor 8) is a zinc-finger transcription factor known to play an essential role in the regulation of the cell cycle, apoptosis, and differentiation. However, its physiological roles and functions in adipogenesis remain unclear. In the present study, we show that KLF8 acts as a key regulator controlling adipocyte differentiation. In 3T3-L1 preadipocytes, we found that KLF8 expression was induced during differentiation, which was followed by expression of peroxisome proliferator-activated receptor γ (PPARγ) and CCAAT/enhancer-binding protein α (C/EBPα). Adipocyte differentiation was significantly attenuated by the addition of siRNA against KLF8, whereas overexpression of KLF8 resulted in enhanced differentiation. Furthermore, luciferase reporter assays demonstrated that overexpression of KLF8 induced PPARγ2 and C/EBPα promoter activity, suggesting that KLF8 is an upstream regulator of PPARγ and C/EBPα. The KLF8 binding sites were localized by site mutation analysis to −191 region in C/EBPα promoter and −303 region in PPARγ promoter, respectively. Taken together, these data reveal that KLF8 is a key component of the transcription factor network that controls terminal differentiation during adipogenesis.

## Introduction

Obesity is the main cause of metabolic syndrome and leads to various complications, including an increased risk of diabetes and cardiovascular diseases [1]. Obesity is characterized by increased adipose tissue mass due to increased adipocyte number (hyperplasia) and increased adipocyte size (hypertrophy) [2]. The number of adipocytes is determined to a large degree by the adipocyte differentiation process, which generates mature adipocytes from fibroblast-like preadipocytes. Therefore, understanding the regulatory processes involved in adipocyte differentiation may help to limit obesity and its pathological consequences.

Adipocyte differentiation is influenced by endocrine and autocrine factors that promote or constrain adipogenesis by intracellular mechanisms that induce the synthesis and activation of adipogenic transcription factors [3]. Murine 3T3-L1 cells differentiate into mature adipocytes when treated with serum-containing medium supplemented with 1-methyl 3-isobutylxanthine, dexamethasone, and insulin. After hormonal induction, growth-arrested 3T3-L1 preadipocytes reenter the cell cycle for additional two rounds of division, known as mitotic clonal expansion, and then express genes required for the adipocyte phenotype [4], [5], [6]. Several transcription factors are involved in adipocyte differentiation. These include CCAAT/enhancer-binding proteins (C/EBPs) [7], [8] and peroxisome proliferator-activated receptor γ (PPARγ) [9], [10]. Both C/EBPβ and C/EBPδ are induced immediately [11] and stimulate cell proliferation and expression of PPARγ and C/EBPα [12]. PPARγ and C/EBPα serve as pleiotropic transcriptional activators that coordinately induce the expression of adipocyte-specific genes that lead to formation of a mature adipocyte.

Members of the Krüppel-like factor (KLF) family of transcription factors are important regulators of development, growth, differentiation, and a number of other physiological cellular processes [13]. The KLF family is composed of at least 17 transcription factors that share homology in their tandem three C2H2 zinc-fingers near their C-terminus [14]. In adipogenesis, KLF family transcription factors act as both activators and repressors in the transcriptional cascade. KLF5, for example, is induced at an early stage of differentiation and activates the PPARγ2 promoter in concert with C/EBPβ [15]. Moreover, KLF4 acts as a regulator at an earlier stage of differentiation by binding to the C/EBPβ promoter together with Krox20, thereby inducing adipocyte differentiation [16]. In contrast, KLF3 represses adipocyte differentiation by recruiting C-terminal binding protein (CtBP) corepressors [17]. Other KLF family proteins have also been proven to promote or inhibit adipocyte differentiation [18].

In the present study, we analyzed the expression patterns of KLF family proteins during 3T3-L1 differentiation using microarray and found that KLF8 was significantly induced after the mitotic clonal expansion period. Adipocyte differentiation was significantly attenuated by KLF8 knockdown, whereas overexpression of KLF8 resulted in enhanced differentiation. Furthermore, overexpression of KLF8 induced PPARγ2 and C/EBPα transcriptional activities, as shown by luciferase assay, suggesting that KLF8 is an upstream regulator of PPARγ and C/EBPα. These results indicate that KLF8 plays an important role in early adipocyte differentiation.

## Materials and Methods

### Cell Isolation from Mice and Ethic Statement

The stromal vascular fraction (SVF) and fat fraction were isolated from mouse epididymal fat pads of 12-week-old mice by digestion with type I collagenase as described [19]. All procedures were approved by the Committee on Animal Investigations of the Yonsei University.

### Cell Culture and in vitro Differentiation

Methods for maintenance and induction of differentiation of 3T3-L1 preadipocytes have been described previously [20]. Briefly, 3T3-L1 preadipocytes were maintained in Dulbecco’s modified Eagle’s medium (DMEM) containing 100 U/ml penicillin, 100 µg/ml streptomycin, and 8 µg/ml biotin, supplemented with 10% heat-inactivated calf serum at 37°C, in an atmosphere of 90% air and 10% CO_2_. To induce differentiation, 2-day postconfluent 3T3-L1 cells (designated day 0) were incubated in DMEM containing 10% FBS, 0.5 mM 3-isobutyl-1-methylzanthine, 1 µM dexamethasone, and 1 µg/ml of insulin (MDI) for 2 days. Cells were then cultured in DMEM containing 10% FBS and insulin for another 2 days, after which they were grown in DMEM containing 10% FBS. Cell numbers were determined on day 2, and oil red-O staining was performed on day 8.

### Western Blot Analysis

At each time indicated, cells were washed in ice-cold phosphate-buffered saline (PBS) and lysed in a buffer containing 1% SDS and 60 mM Tris-Cl, pH 6.8. Lysates were briefly vortexed, boiled for 10 min, and cleared by centrifugation at 12,000 g for 10 min at 4°C. The supernatants were collected, and protein concentrations were measured using a BCA assay kit (Pierce). Protein samples of equal amount were separated by SDS-PAGE and transferred to nitrocellulose membranes. Anti-KLF8 rabbit polyclonal antibody was generated against the murine KLF8 peptide (residues 5–16, IDNMDVRIKSES) by Atigen (Korea). Immunoblot analyses were performed using the following antibodies: polyclonal antibody against KLF8, C/EBPα [21], C/EBPβ [19], mouse monoclonal antibodies against PPARγ (Santa Cruz Biotechnology), FLAG antibody (Sigma), and β-actin antibody (Santa Cruz Biotechnology). The immunoreactive bands were detected using an enhanced chemiluminescence detection system (Amersham) following the manufacturer’s instructions.

### RNA Isolation and Real-time RT-PCR

Total RNA was isolated from cultured cells using TRIzol (Invitrogen) according to the manufacturer’s instructions. For quantitative RT-PCR, cDNA was synthesized from 5 µg of total RNA using random hexamer primers and SuperScript reverse transcriptase II (Invitrogen), following the manufacturer’s instructions. An aliquot (1/40) of the reaction was used for quantitative PCR using the SYBR Green PCR Master Mix (Applied Biosystems) and gene-specific primers. RT-PCR products were quantified using the ABI PRISM 7300 RT-PCR System (Applied Biosystems). RT-PCR was performed using the following primers: KLF8-sense, 5′-CAAGC CATTA TGGTG CCTAC-3′; KLF8-antisense, 5′-ATAGA GCCCG GAGTG AGAAC-3′. All reactions were performed in triplicate. The relative amounts of the mRNA were calculated using the comparative cycle-time method (Applied Biosystems). GAPDH mRNA was also measured as an invariant control.

### Small Interfering RNA (siRNA)

Preadipocytes (3T3-L1) were plated into 60-mm-diameter dishes 18–24 h prior to transfection. Cells were transfected with control or gene-specific siRNA at 50 nM (Dharmacon) in OPTI-MEM medium using Lipofectamine RNAiMAX (Invitrogen), according to the manufacturer’s protocol. The next day, the medium was replaced with fresh DMEM containing 10% calf serum and the cells were incubated for 24 h before the induction of differentiation. Total RNA and protein extracts were prepared from the cells at the indicated time points, and RT-PCR and immunoblot analyses were performed. Oil red-O staining of KLF8 knockdown was performed at day 8. The siRNA sequences are as follows: si-C/EBPβ, 5′-AGUAG AAGUU GGCCA CUUCC AUGGG-3′; si-KLF8a, 5′-UGAAG UAGGC ACCAU AAUGG CUUGA -3′; and si-KLF8b, 5′-UCAAG CCAUU AUGGU GCCUA CUUCA-3′. We used the Stealth RNA siRNA negative control (Invitrogen) as si-RNA control.

### Transient Transfection Assay

KLF8 overexpressing vector (pcDNA3.0-KLF8-FLAG) was generated by inserting the whole open reading frame of mouse KLF8 with a C-terminal FLAG tag into pcDNA3.0 (Invitrogen). To maximize the transfection efficiency, microliter volume electrophoration of 3T3-L1 preadipocytes was performed with OneDrop MicroPorator MP-100 (Digital Bio). The cells were trypsinized, washed with 1× PBS, and finally resuspended in 10 µl of resuspension buffer R and 0.5 µg of plasmid at a concentration of 200,000 cells per pipette. The cells were then microporated at 1,300 V, with a 20-ms pulse width, 2 pulses. Following microporation, the cells were seeded in 35-mm cell culture dishes and placed at 37°C in a 10% CO_2_-humidified atmosphere. For luciferase assays of the promoter constructs, Lipopfectamin and Plus Reagent (Invitrogen) was used. Briefly, NIH3T3 cells were cultured at a density of 2.5×10^5^ cells/well in DMEM. The next day, cells were transfected with the indicated luciferase reporter plasmids using Lipofectamine and Plus Reagent following the manufacturer’s instructions. After 3 h of incubation, the cell medium was replaced with fresh complete medium. After 48 h of incubation, the cells were washed with PBS and harvested in 200 µl of passive lysis buffer (Promega). The cells were mixed vigorously for 15 s and centrifuged at 12,000 g for 10 min at 4°C. The supernatants were transferred into a fresh tube, and 5-µl aliquots of the cleared whole-cell lysate were assayed for luciferase activity using a Dual-Luciferase Reporter Assay System (Promega). Each transfection experiment was performed in triplicate.

### Site-directed Mutagenesis

PCR amplification of the wild-type luciferase reporter plasmid was performed using site-directed mutation primers (C/EBPαmt-C/EBP-sense 5′-AGCGC AGGAG TCAGT GGGCG TTGat aCACG ATCTC-3′, C/EBPαmt-C/EBP-antisense 5′-GAGAT CGTGt atCAA CGCCC ACTGA CTCCT GCGCT-3′; C/EBPαmt-KLF-sense 5′-AGCGC AGGAG TCAGT GGtgt TTGCG CCACG ATCTC-3′, C/EBPαmt-KLF-antisense 5′-GAGAT CGTGG CGCAA acaCC ACTGA CTCCT GCGCT-3′, PPARγ2mtKLF-sense 5′-AACTA CTGTA CAGTT acaGC CCCTC ACAGA-3′, PPARγ2mt-KLF-antisense 5′-TCTGT GAGGG GCtgt AACTG TACAG TAGTT-3′). The substituted bases are indicated in small case letter. PCR amplification was performed using 50 ng template DNA and 15 cycles of 95°C for 1 min, 55°C for 1 min and 72°C for 7 min. PCR products were digested with *Dpn*I for 2 h at 37°C, prior to transformation into *Escherichia coli* DH5α competent cells. Colonies were screened by DNA sequencing.

### Electrophoretic Mobility Shift Assays (EMSA)

Briefly, pcDNA3.0-C/EBPβ-FLAG and KLF8-FLAG were used for in vitro-translation reaction using TNT T7 quick master mix (Promega). EMSAs were performed using in vitro-translated protein as previously described [21]. Double-stranded probes were labeled with [γ-^32^P]ATP, using T4 polynucleotide kinase. Protein-DNA complexes were resolved from the free probe by electrophoresis on a 4%(wt/vol) polyacrylamide gel in 0.25× TBE buffer. The dried gels were exposed to X-ray film with an intensifying screen. Probe sequences are as follows; β-globin, sense 5′-TAGAG CCACA CCCTG GTAAG-3′, antisense 5′-CTTAC CAGGG TGTGG CTCTA-3′, C/EBPα, sense 5′-AGCGC AGGAG TCAGT GGGCG TTGCG CCACG-3′, antisense 5′-CGTGG CGCAA CGCCC ACTGA CTCCT GCGCT-3′, PPARγ2, sense 5′-CTGTA CAGTT CACGC CCCTC ACAGA-3′, antisense 5′-TCTGT GAGGG GCGTG AACTG TACAG-3′. Also, mutated probe sequences were used as described in site-directed mutagenesis.

### Chromatin Immunoprecipitation (ChIP) Assays

ChIP analysis was performed following the protocol of the ChIP assay kit (Upstate). DNA-protein complexes were immunoprecipitated with antibodies against C/EBPβ and KLF8 for 4 h and then collected with protein A-agarose for 3 h at 4°C with rotation. The beads were washed, and chromatin complexes were eluted from the beads. After reversal of the cross-links, the DNA was purified. Input control and ChIP samples were used as PCR templates to amplify the PPARγ and C/EBPα promoters containing the C/EBP and KLF8 binding sites using the following primers: C/EBPα, the region from −335 to −83 was amplified, sense 5′-TCCCT AGTGT TGGCT GGAAG-3′, antisense 5′-CAGTA GGATG GTGCC TGCTG-3′, PPARγ2, the region from −618 to −119 was amplified, sense 5′-ATTTA AATTT TACTA GCCTT-3′, antisense 5′- GACAA AATGG TGTGT CATAA-3′.

### Statistical Analysis

All results are expressed as mean ± SD. Statistical comparisons of groups were made using an unpaired Student’s t test and two-way ANOVA.

## Results

### KLF8 is Induced After Mitotic Clonal Expansion in 3T3-L1 Cell Differentiation

In order to analyze the roles of KLF family proteins in adipogenesis, we conducted microarray during 3T3-L1 cell differentiation induced by the standard hormone cocktail (MDI) (Table 1). In this analysis, it is remarkable that KLF8, KLF9, KLF12, and KLF17 were induced during adipocyte differentiation. Interestingly, the expression pattern of KLF8, KLF12, and KLF17 were similar, as they reached peak expression at day 2 after differentiation induction, suggesting that these KLFs may play roles in terminal differentiation during adipogenesis.

**Table 1:** Gene expression change of KLF family members during 3T3-L1 differentiation.

			mRNA expression fold			
Gene	Alternative Names	GenBank No.	D0	D2	D4	D7
Klf1	Eklf	NM_010635	1	0.916	1.020	1.233
Klf2	Lklf	NM_008452	1	1.195	1.166	1.187
Klf3	Bklf; Tef-2	NM_008453	1	0.800	1.291	0.852
Klf4	EZF; Zie; Gklf	NM_010637	1	0.404	0.352	0.341
Klf5	CKLF; IKLF; Bteb2	NM_009769	1	1.544	0.858	0.584
Klf6	FM2; FM6; Zf9	NM_011803	1	0.271	0.547	0.546
Klf7	–	NM_033563	1	1.642	0.643	0.621
Klf8	BKLF3; ZNF74	NM_173780	1	2.417	1.928	1.830
Klf9	Bteb1; BTEB-1	NM_010638	1	2.160	4.008	3.286
Klf10	Tieg; mGIF; Egral; Tieg1	NM_013692	1	1.088	0.670	0.621
Klf11	Tieg2; Tieg3; Tieg2b	NM_178357	1	0.288	0.348	0.686
Klf12	AP-2rep	NM_010636	1	3.108	1.765	1.167
Klf13	Bteb3; FKLF2; NSLP1	NM_021366	1	1.796	1.702	3.855
Klf16	DRRF; BTEB4	NM_078477	1	1.516	0.736	1.142
Klf17	Gzf; C85123; Zfp393	NM_029416	1	2.489	1.631	0.858

* Microarray was performed using total RNA samples from 3T3-L1 cells after 0, 2, 4, and 7 days of differentiation. Gene expression was analyzed by Agilent Mouse Genome 4×44 k oligo chip. The preparation for hybridization and the scanning of mouse chips were performed according to the manufacturer’s protocols (Genocheck). More than 2-fold changes of expression are indicated in bold.

**Figure 1:**
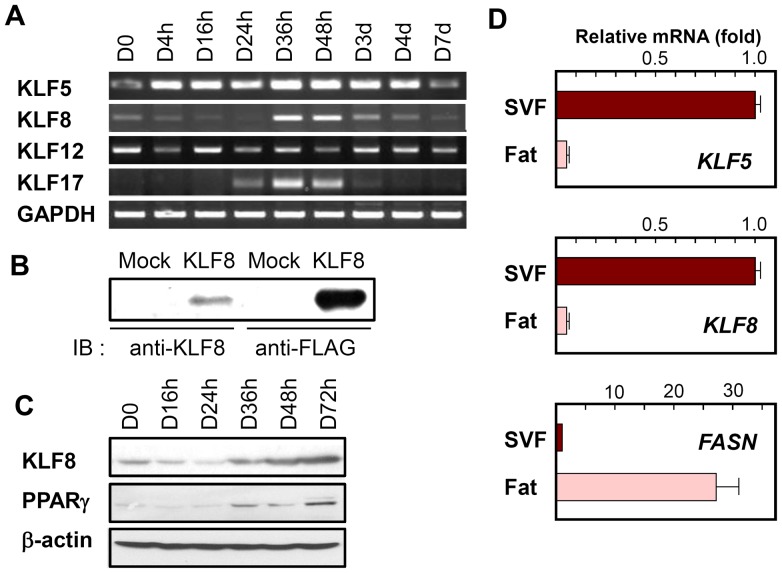
KLF8 expression during 3T3-L1 adipocyte differentiation and in mouse adipose tissue. (A) Total RNA was extracted from 3T3-L1 cells at the indicated times before and after induction of differentiation. The expression levels of KLF5, KLF8, KLF12, and KLF17 were determined by RT-PCR. (B) Anti-rabbit polyclonal KLF8 antibody was generated and tested on 293T cells that were transfected with either pcDNA3.0 or pcDNA3.0-KLF8-FLAG. Whole-cell lysates were immunoblotted (IB) using anti-KLF8 or anti-FLAG, which verified the specific antigen-antibody interaction. (C) Western blot analysis of KLF8 expression was performed at the indicated time points during 3T3-L1 cell differentiation. (D) KLF5, KLF8, and fatty acid synthase (FASN) mRNA levels were measured in the stromal vascular fraction (SVF) or the fat fraction of mouse epididymal adipose tissue using real-time qPCR. Data represent the mean ± SD.

To confirm the microarray data, the expression levels of these KLFs were analyzed by RT-PCR at different time points. As shown in Figure 1A, the amount of KLF8 mRNA was increased during 3T3-L1 differentiation, reaching its maximum at 36–48 h after induction. In contrast, the mRNA level of KLF5 was elevated at an earlier point during differentiation. Similar to KLF8, KLF17 also was upregulated at the mRNA level during 3T3-L1 differentiation. Contrary to the microarray data, however, KLF12 was not found to be induced during differentiation by RT-PCR. Therefore, we focused on the potential roles of KLF8 and KLF17 in the differentiation program of adipogenesis. We generated polyclonal anti-KLF8 antibody (Figure 1B) and verified the increase of KLF8 protein during adipocyte differentiation (Figure 1C), suggesting that KLF8 is involved in the terminal differentiation of adipocytes. It is interesting to note that KLF8 was mainly expressed in the stromal vascular fraction (SVF) compared to the fat fraction (Figure 1D). The SVF is known to have many adipocyte progenitor cells [22], whereas the fat fraction contains mature adipocytes. This suggests that KLF8, like KLF5, which is also expressed in the SVF, is not required for maintenance of the mature adipocyte phenotype, but instead plays a role in the differentiation of adipocytes.

### KLF8, but not KLF17, is Necessary for Adipocyte Differentiation in 3T3-L1 Preadipocytes

**Figure 2:**
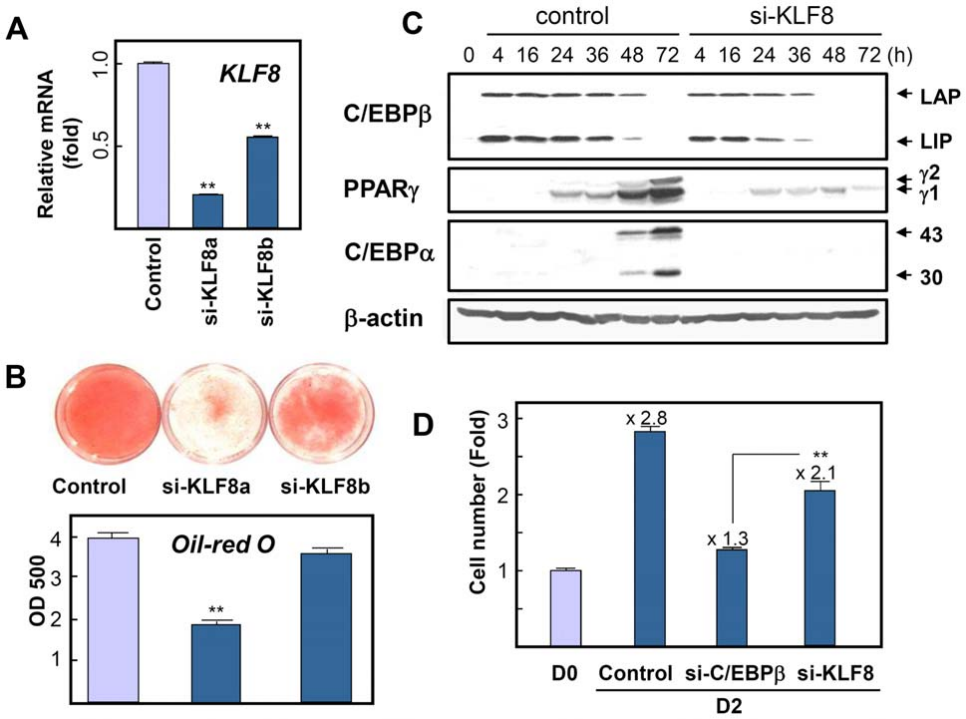
KLF8 knockdown blocks 3T3-L1 adipocyte differentiation. Preadipocyte 3T3-L1 cells were treated with KLF8 siRNA at about 70% confluence using Lipofectamine RNAi/MAX reagent. After 24 h, the cells were trypsinized and replated at confluent cell density. After an additional 24 h, the cells were induced to differentiate. (A) Twenty hours after induction, cell extracts were prepared, and the effect on the expression of KLF8 was analyzed by real-time RT-PCR. (B) Oil red-O staining on day 8. The low panel represents spectrophotometric count of staining from 3 independent experiments. (C) Cells were harvested at the indicated times, and cell lysates were separated by SDS-PAGE and immunoblotted with antibody against C/EBPβ, PPARγ, and C/EBPα. LAP, liver-activating protein, LIP, liver-inhibitory protein, PPARγ1, PPARγ2, and 43 or 30 kDa of C/EBPα proteins were indicated. (D) To investigate the effect of siRNA on mitotic clonal expansion, cell numbers were determined at day 0 or day 2 after induction. Fold increases compared to the cell number in D0 were marked. Data in (A), (B), and (D) represent the mean ± SD. **P<0.01.

To confirm the function of KLF8 directly, we observed the effect of KLF8 knockdown in 3T3-L1 preadipocytes. Two KLF8-specific siRNAs (designated as si-KLF8a and b) were used in 3T3-L1 cell transfection prior to hormonal induction of differentiation. The mRNA levels of KLF8 decreased approximately to 75% and 40% by si-KLF8a and b, respectively, compared to control siRNA (Figure 2A). Importantly, oil red-O staining on day 8 showed that KLF8 siRNA significantly diminished the accumulation of lipid droplets (Figure 2B). Moreover, the expression levels of PPARγ and C/EBPα were effectively suppressed by KLF8 siRNA during differentiation compared to expression levels in the control siRNA group, whereas C/EBPβ expression was relatively unaffected (Figure 2C). This suggests that KLF8 acts as an upstream regulator of C/EBPα and PPARγ, independent of C/EBPβ. Meanwhile, although the expression of KLF17 was induced during differentiation, knockdown of KLF17 did not affect 3T3-L1 cell differentiation (data not shown).

While expression of C/EBPβ increases after induction of differentiation, it has been reported that the upregulation of C/EBPα is delayed [23], suggesting the presence of another regulator between the two transcription factors. Because C/EBPβ is also required for mitotic clonal expansion [24], knockdown of C/EBPβ resulted in the inhibition of cell proliferation (Figure 2D). Meanwhile, cell numbers at day 2 indicate that knockdown of KLF8 did not inhibit mitotic clonal expansion to the level observed in si-C/EBPβ (Figure 2D), suggesting that the role of KLF8 is downstream of C/EBPβ and mitotic clonal expansion. This is consistent with the result that expression of KLF8 increases at 36–48 h after the first round of division during clonal expansion (Figure 1A). Thus, KLF8 might not affect cell cycle or the expression of C/EBPβ but play a critical role in terminal differentiation during adipogenesis.

**Figure 3:**
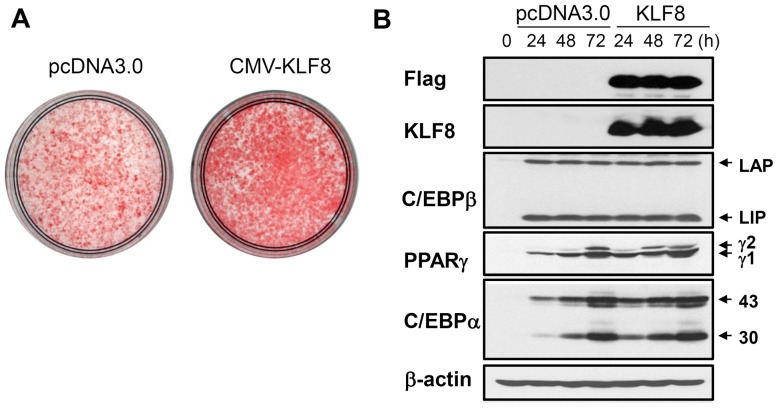
Overexpression of KLF8 results in enhanced differentiation. Preadipocyte 3T3-L1 cells were transiently transfected using electroporation with pcDNA3.0-KLF8-FLAG or empty vector and differentiated with standard hormonal cocktail as described in Materials and Methods. (A) After 5 days of differentiation, lipid accumulation was detected by oil red-O staining. (B) Western blot analysis of whole-cell extracts prepared at the indicated times during differentiation. LAP, liver-activating protein, LIP, liver-inhibitory protein, PPARγ1, PPARγ2, and 43 or 30 kDa of C/EBPα proteins were indicated.

We next examined whether overexpression of KLF8 might induce adipogenesis in 3T3-L1 cells. KLF8 was overexpressed in 3T3-L1 preadipocytes by microporation prior to hormonal induction of differentiation. As expected, KLF8 overexpression increased the accumulation of lipid droplets (Figure 3A). In addition, PPARγ and C/EBPα were induced earlier in KLF8-overexpressing cells than in control cells, whereas no difference was observed in the induction of C/EBPβ (Figure 3B). Thus, it appears that KLF8 is able to induce the expression of PPARγ and C/EBPα.

### KLF8 Directly Controls and Binds to the PPARγ2 and C/EBPα Promoters

**Figure 4:**
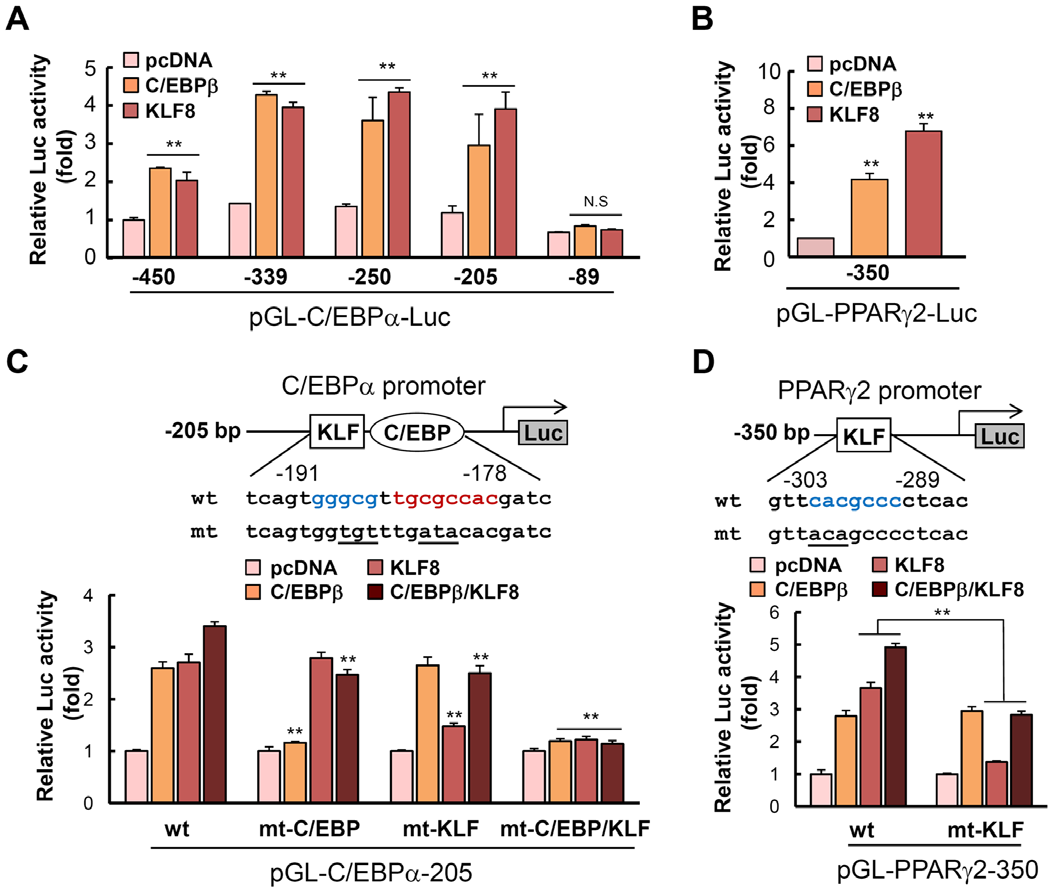
KLF8 regulates C/EBPα and PPARγ2 promoters to induce transcription. (A) A series of C/EBPα promoter constructs cloned into the pGL3-Basic vector was used in this study. NIH3T3 cells were transfected with the luciferase constructs using Lipofectamine, and the luciferase activities were determined after 2 days. The plasmids pcDNA3.0, pCMV-C/EBPβ, or pCMV-KLF8-FLAG were cotransfected. (B) Similarly, the PPARγ2 promoter (−350 counted from transcription initiation site) was investigated by luciferase assay. (C) Mutation analysis of the C/EBPα promoter was performed. The −205 construct was used for the site directed mutagenesis in order to introduce KLF and/or C/EBP regulatory element between −191 and −178 region. The KLF8 binding site was indicated in blue, whereas the C/EBP site was marked in red. The mutated region was underlined. NIH3T3 cells were transfected with these constructs along with KLF8 and/or C/EBPβ overexpression plasmids. (D) Mutation analysis of the PPARγ2 promoter was performed. The KLF site was mutated by site-specific mutagenesis, and the resulting construct was analysed by luciferase assay. Data represent the mean ± SD. **P<0.01.

Because we found that KLF8 plays an important role in PPARγ and C/EBPα expression, we next examined whether KLF8 directly controls transcription of these genes. To clarify which region is responsible for KLF8 transactivation, serial deletion constructs of a −450 fragment of the C/EBPα promoter were made [25]. KLF8, similar to C/EBPβ, significantly stimulated C/EBPα promoter activity in reporter assays (Figure 4A). This activation was almost completely abolished when the minimal proximal promoter (C/EBPα-89) was used (Figure 4A), indicating that KLF8 plays a critical role in C/EBPα promoter activity and the direct binding site exists between −205 and −89 bp in the C/EBPα promoter. Also, we found that KLF8 stimulated PPARγ promoter activity directly (Figure 4B). Sequence analysis of the C/EBPα promoter revealed two potential binding sites for KLF family members, the consensus sequence for which has been identified as CNCCC [26]. Of these, site-specific mutation of the −191 KLF site abolished luciferase activity driven by KLF8 overexpression (Figure 4C). It should be noted that the KLF8 binding site is a GC-rich region right next to the C/EBP regulatory element [7]. The overexpression of both C/EBPβ and KLF8 resulted in the activation of the C/EBPα-205-luciferase construct in a partially additive manner, not synergistically. Consistently, the mutation study revealed that C/EBPβ and KLF8 bind to the C/EBPα promoter separately, without affecting the action of the other (Figure 4C). Similarly, KLF8 site was localized to −303 region of the PPARγ2 promoter, which has been identified as a KLF5 binding site previously [15] (Figure 4D). Mutation of this sequence also abolished KLF8-driven transactivation of the promoter, without affecting C/EBPβ action.

**Figure 5:**
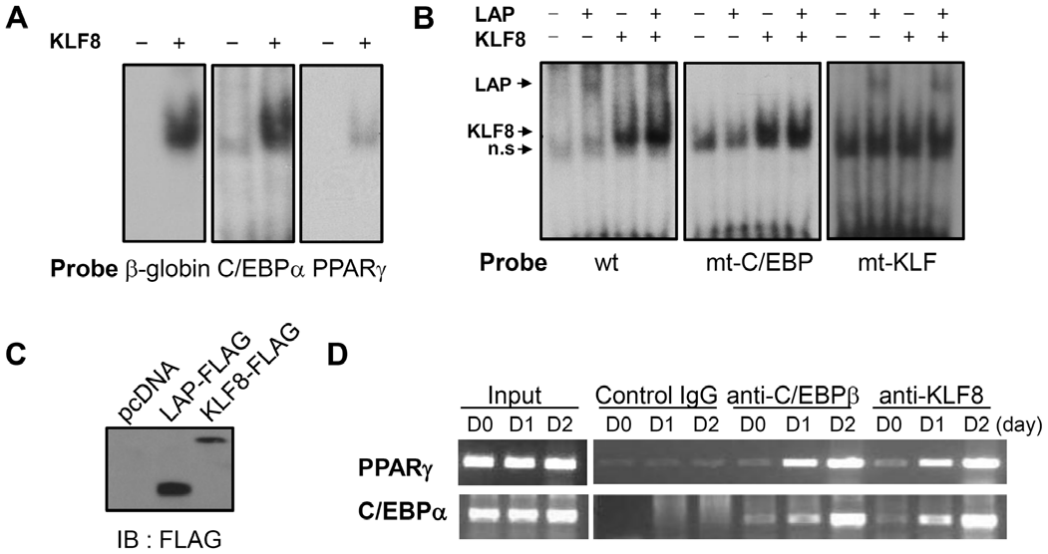
KLF8 directly binds to C/EBPα and PPARγ2 promoter. (A) EMSA experiment using KLF binding sites of β-globin (positive control), C/EBPα, or PPARγ2 promoter as probes. A FLAG-tagged KLF8 protein was in vitro translated using TNT T7 quick master mix. (B) EMSA experiment using wild type or mutated probe of the C/EBPα promoter. FLAG-tagged C/EBPβ-LAP or KLF8 protein was in vitro translated using TNT T7 quick master mix. (C) Western blot analysis of the in vitro translated proteins. n.s, non-specific. (D) ChIP was performed on 3T3-L1 cell chromatin at the indicated time points after induction of differentiation, using control IgG, anti-C/EBPβ, or anti-KLF8 antibody. The immunoprecipitated DNA was used as a PCR template to detect the PPARγ or C/EBPα promoter regions.

The direct binding of KLF8 to the promoters was also confirmed by EMSA. As shown in Figure 5A, in vitro translated KLF8 bound to −191 region of the C/EBPα promoter and −303 region of the PPARγ2 promoter, respectively. A known sequence of the β-globin promoter was shown as a positive control. As mentioned above, −191 region of the C/EBPα promoter has an interesting feature that KLF8 and C/EBPβ binding sites exist in a short sequence. Thus, we tested whether KLF8 and C/EBPβ bind separately using mutated probes. The Figure 5B and 5C show that KLF8 or C/EBPβ binding was not basically affected by each other factor. This is consistent with the luciferase result: KLF8 and C/EBPβ transactivate the promoter in an additive manner (Figure 4C).

To further confirm the role of KLF8 on these promoters, we next performed ChIP to analyze the binding of KLF8 to these target genes. Chromatin samples were prepared from 3T3-L1 cells before (day 0) and after the induction of adipocyte differentiation (days 1 and 2) and were then immunoprecipitated with a KLF8-specific antibody. We found that KLF8 bound to these promoters (day 1) and the strength of binding increased further after 2 days of MDI treatment (Figure 5D). Anti-C/EBPβ and anti-IgG were used as positive and negative control, respectively. Taken together, the results of this study implicate KLF8 as a key component of the transcription factor network that controls the regulation of C/EBPα and PPARγ during adipogenesis.

## Discussion

In this study, we showed that KLF8 expression increased at both the mRNA and protein level during adipocyte differentiation in 3T3-L1 cells. In addition, KLF8 controlled the expression of PPARγ and C/EBPα by directly binding to the promoter regions but had no effect on C/EBPβ. These results suggest that KLF8 is a key intermediate component of the transcription factor network regulating adipogenesis.

Previous studies identified that differentiation of the 3T3-L1 preadipocyte cell line into adipocytes requires sequential activation of various transcription factors, including C/EBPβ, C/EBPδ, PPARγ, and C/EBPα [4]. C/EBPβ and C/EBPδ are expressed in the early stage of adipogenesis (at ∼4 h), whereas the activation of the C/EBPα or PPARγ gene is initiated ∼36 h after differentiation is induced. Therefore, it is suggested that additional events are required for the terminal differentiation. The phosphorylation-dependent activation process of C/EBPβ by MAPK and GSK3β [21], [27], as well as redox control of DNA-binding [19] were suggested for those events. In addition, we also reported that upstream stimulatory factors are required for the full activation of C/EBPα promoter [25].

Meanwhile, the involvement of KLF family proteins provides another example for the completion of transcriptional cascade in adipogenesis. KLF family proteins are known to play diverse roles in cell differentiation and development in mammals. Although KLF proteins exhibit homology in their carboxyl-terminal zinc finger domains, different amino-terminal sequences provide unique regions for interaction with specific binding partners. It is well established that KLF8 acts in critical cellular processes, such as differentiation, cell cycle progression, transformation, epithelial-to-mesenchymal transition, migration, and invasion [14]. In the present study, we first report the involvement of KLF8 in the transcriptional cascade of adipocyte differentiation. Importantly, KLF8 was expressed at ∼36 h just prior to the activation of C/EBPα and PPARγ promoter. In addition, KLF8 overexpression in 3T3-L1 cells strongly upregulated PPARγ and C/EBPα expression by directly binding to these gene promoters. Furthermore, our analysis showed that the role of KLF8 is initiated after mitotic clonal expansion, suggesting that KLF8 is a critical regulator of terminal differentiation in adipogenesis.

We found that KLF8 binding site on the C/EBPα promoter is a GC-rich region right next to the C/EBP regulatory element. This GC-rich region has been analyzed thoroughly, demonstrating that Sp1 occupies the GC-box, which prevents access of the C/EBP protein in preadipocytes [28]. Upon differentiation stimuli, Sp1 level is decreased and C/EBP then binds to the regulatory region, thereby activating the C/EBPα promoter activity [28]. In our data, KLF8 and C/EBPβ bind to and activate the C/EBPα promoter, in an additive manner. We also tested whether these two transcription factors interact with each other by protein-protein interaction; however, we could not observe any detectible interaction by immunoprecipitation (data not shown). Therefore, we suggest a possible mechanism involved in an activation process of the C/EBPα promoter as follows; in preadipocytes, Sp1 expression is high enough to repress the C/EBPα promoter, by competing the regulatory element with C/EBPβ. When the differentiation is induced, the cells down-regulate Sp1 and begin to express C/EBPβ and KLF8. These two transcription factors occupy the critical regulatory elements of the C/EBPα promoter, thereby leading to a steady expression of C/EBPα protein. It is also possible that this combination of KLF8 and C/EBPβ recruits specific co-activators to the promoter region. In previous studies, KLF8 has been found to recruit p300/CBP in order to activate expression of the cyclin D1 gene and promote acetylation of nearby histones [29]. Post-translational modification of KLF8 via SUMOlyation attenuates this ability [30]. Whether recruitment of p300/CBP or post-translational modification of KLF8 are involved in adipogenesis needs to be further investigated.

Recently, it was reported that the closely related family member KLF3 is highly expressed in undifferentiated preadipocytes and reduced upon differentiation into adipocytes [17]. Other study demonstrated that KLF3 repressed expression of KLF8 in other cell types [31], raising a possibility of network between KLF family of proteins during adipocyte differentiation. In this regard, it is possible that the activities of KLF3 and KLF8 are reciprocally regulated during adipogenesis. Thus, it would be interesting to investigate the KLF family protein network in adipogenesis, including regulation of the KLF3-KLF8 axis.

In summary, the present study provides interesting evidence for the pivotal role played by KLF8 in adipocyte differentiation. Further studies of the mechanisms by which KLF8 expression and function are regulated should provide additional insight into adipocyte differentiation.
